# Accurate and precise in vivo liver 3D *T*_1_ mapping at 3T

**DOI:** 10.1002/mrm.30448

**Published:** 2025-02-04

**Authors:** Gabriela Belsley, Ferenc E. Mózes, Damian J. Tyler, Matthew D. Robson, Elizabeth M. Tunnicliffe

**Affiliations:** ^1^ Oxford Centre for Clinical Magnetic Resonance Research, Radcliffe Department of Medicine University of Oxford Oxford UK; ^2^ Perspectum Oxford UK

**Keywords:** 3D $T_{1}$ mapping, accuracy, liver, precision, quantitative MRI

## Abstract

**Purpose:**

To develop an accurate and precise liver 3D $T_{1}$ mapping method using only scanner‐agnostic sequences.

**Methods:**

While the spoiled gradient‐recalled echo sequence is widely available on clinical scanners, variable flip angle $T_{1}$ mapping methods based on this sequence provide biased $T_{1}$ estimates, with the largest systematic error arising from $B_{1}^{+}$ inhomogeneities. To correct for this, the flip angle was mapped using a 2D gradient‐echo double‐angle method approach. To correct for the confounding effect of fat on liver $T_{1}$ and $B_{1}^{+}$, Dixon and fat saturation techniques were used in combination with the variable flip angle and the $B_{1}^{+}$ map acquisitions, respectively. The $T_{1}$ and $B_{1}^{+}$ mapping methods were validated with a $T_{1}$‐phantom against gold standard methods. An intra‐ and inter‐repeatability study was conducted at 3T in 10 healthy individuals' livers.

**Results:**

The developed 3D $T_{1}$ mapping method achieved an excellent agreement with the gold standard, with a weighted root mean squared normalized error below 2.8%. In vivo, a median $T_{1}$ standard deviation of 31 ms and an interquartile range of [27, 39] ms was achieved across all measurements, including the intra‐ and inter‐repeatability study data. A within‐subject standard deviation for $T_{1}$ of 21 ± 5 ms had a corresponding repeatability coefficient of 60 ms. The measured $T_{1}$ values agree well with MOLLI and SASHA $T_{1}$ mapping methods, with average $T_{1}$ differences of 5%.

**Conclusion:**

Accurate and precise 3D $T_{1}$ liver measurements can lead the way to the wider adoption of a clinically feasible $T_{1}$ measurement as a marker of hepatic fibro‐inflammation.

## 1 INTRODUCTION

Quantitative MRI liver studies have shown that non‐contrast liver $T_{1}$ may be a promising novel biomarker for aiding in the diagnosis, staging, monitoring, and prognosis of liver diseases as increases in liver $T_{1}$ correlate with liver fibrosis and inflammation.^1^, ^2^, ^3^ $T_{1}$ mapping has been carried out on patients, spanning pediatric subjects to elderly cohorts, with different liver etiologies including metabolic dysfunction associated steatotic liver disease (MASLD),^1^, ^2^ metabolic dysfunction associated steatohepatitis (MASH),^4^ cirrhosis,^5^, ^6^ chronic liver diseases,^7^, ^8^ portal hypertension,^9^, ^10^ hepatocellular carcinoma (HCC),^11^, ^12^, ^13^ and autoimmune liver diseases.^14^, ^15^

The adoption of new quantitative methods in clinical practice requires high repeatability and reproducibility. Repeatability represents the agreement between replicate measurements over a short period of time under a set of conditions.^16^ The modified Look‐Locker inversion recovery (MOLLI),^17^, ^18^ saturation recovery single‐shot acquisition (SASHA),^19^, ^20^ and inversion recovery spin echo echoplanar imaging (IR‐SE‐EPI)^3^, ^5^ $T_{1}$ mapping sequences were shown to be repeatable both in vitro and in vivo. Despite the repeatability of MOLLI and SASHA, these methods are restricted to imaging a single slice within a breath‐hold. Whole liver $T_{1}$ mapping would be advantageous to characterize heterogeneity in fibrosis and inflammation across the entire organ. This could lead to a better diagnosis, understanding, and treatment of liver diseases. The evaluation of focal liver diseases, such as HCC or biliary diseases (e.g., primary biliary cholangitis, primary sclerosing cholangitis) would especially benefit from 3D acquisitions. HCC screening will unquestionably miss tumors if whole liver coverage is not used. The second limitation of MOLLI $T_{1}$ mapping is the requirement of a relatively modern scanner with a dedicated $T_{1}$ mapping license, limiting its deployment. Thirdly, MOLLI $T_{1}$ estimates are biased by magnetisation transfer^21^ and $T_{2}$ effects,^22^ as well as by the presence of iron^23^ and fat.^24^ Moreover, neither SASHA^20^ nor multiple TI 3D IR‐SE‐EPI^3^ are available as product sequences which prevents their immediate clinical use and adoption. Reproducibility of quantitative $T_{1}$ mapping may also be affected by differences in each vendor's implementations of pulse sequences which can result in $T_{1}$ bias. This is the case for MOLLI, where a linear mapping was needed to standardize $T_{1}$ values between two different vendors at the same field strength.^18^

The spoiled gradient recalled echo (SPGR) is a widely available pulse sequence, offering 3D $T_{1}$‐weighted images of the liver. However, when used for $T_{1}$ mapping with a variable flip angle approach (VFA) the sequence is very sensitive to $B_{1}^{+}$ inhomogeneities.^25^, ^26^ The fractional error in $T_{1}$ is equal to the $B_{1}^{+}$ factor squared minus one,^27^ for example, for a $B_{1}^{+}$ factor of 0.6, the relative error in $T_{1}$ would be −64%. Even though it is essential to correct for $B_{1}^{+}$ inhomogeneities to obtain an accurate $T_{1}$, multi‐vendor reproducibility studies tend to skip this extra step due to the lack of a standardized $B_{1}^{+}$ mapping sequence available across all vendors.^28^, ^29^ Fat is another confounding factor of liver $T_{1},$ ^24^, ^30^, ^31^ especially in the case of MASLD and MASH patients. For an unbiased estimate of liver $T_{1}$, water specific VFA contrasts are needed to avoid contamination from the fat signal in each voxel. In this work, we used a VFA SPGR sequence with Dixon (dual echo) fat‐water separation and a gradient‐echo sequence with fat saturation for the $B_{1}^{+}$ map.

The aim of this study was to develop a $T_{1}$ mapping method, covering the majority of the liver volume, that uses sequences widely available on any scanner, independent of the manufacturer. This will facilitate widespread clinical use of the method, removing the need for specialized pulse sequence programming or research agreements, and enabling the comparison of $T_{1}$ values in multi‐center studies, as well as in clinical trials using different scanners in multiple countries. The primary goal of the study was to assess the repeatability of the method in vivo to test whether it is affected by the positioning of the patient in the MRI scanner or from scanner calibration. Our secondary objective was to characterize the in vivo accuracy of the method. These are important steps toward adopting liver $T_{1}$ as a biomarker in clinical practice.

## 2 METHODS

Imaging data were acquired on a 3T Prisma scanner (Siemens Healthineers, Erlangen, Germany), using a spine array coil and an 18‐channel array coil.

### 2.1 Phantom scans

To test the accuracy of the developed $T_{1}$ mapping method, an overnight acquisition using gold standard (GS) $B_{1}^{+}$ and $T_{1}$ mapping sequences were run on a $T_{1}$ phantom with varying $T_{1}s$ and no fat. The GS $B_{1}^{+}$ map consisted of a 3D non‐selective double‐angle method (DAM) gradient‐recalled echo (GRE) acquisition with a long TR of 10 s allowing for full relaxation of longitudinal magnetization. For the GS $T_{1}$ map, a slice‐selective inversion recovery spin echo (IR SE) acquisition was used with TIs logarithmically increasing from 25 to 5000 ms. Other acquisition parameters and details of data processing are reported in the Supporting Information S1. The $T_{1}$ phantom was built by Perspectum Ltd. (Oxford, UK) and consists of 14 vials each of which contains 3% agar gel with NiCl_2_ as the doping agent. This resulted in vials with $T_{1}s$ ranging from 367 ms to 1699 ms (at 3T), in steps of approximately 100 ms and a $T_{2}$ of 45 ms.

### 2.2 In vivo scans

**FIGURE 1:**
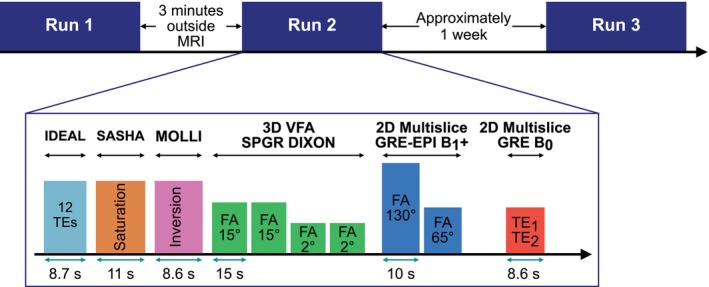
Imaging sequences acquired for the three runs of the healthy volunteer repeatability study.

Data were acquired from 10 healthy volunteers (5 female and 5 male). The study was designed to assess the repeatability of 3D $T_{1}$ mapping, therefore volunteers were scanned over two visits on 2 different days, and twice during visit one. Volunteers were scanned according to our institution's ethical practices and gave informed consent. The image acquisition pipeline is illustrated in Figure 1. Between run 1 and run 2 the volunteer was asked to step out of the scanner, rest for approximately 3 min, and then go back into the scanner to acquire repeated data. In a second visit, approximately 1 week after the first visit, the volunteer was re‐scanned only once with the same sequence scheme. Volunteers food fasted overnight, and water fasted for 30 min before their scan in the morning, as Mózes et al.^32^ has shown a dependence of liver shMOLLI $T_{1}$ values on glycogen and hydration levels. Images for all sequences were acquired in the transverse plane with phase‐encoding in the anterior posterior direction.

$T_{2}^{*}$ and proton density fat‐fraction (PDFF) were calculated from a LiverMultiScan (Perspectum Ltd., Oxford, UK) multi‐echo $T_{2}^{*}$‐Iterative Decomposition of Water and Fat with Echo Asymmetry and Least‐Squares Estimation (IDEAL)^33^ protocol with imaging parameters: FA = 3°, TR = 15 ms, 12 TEs (1.1–13.2 ms, incremented in steps of 1.1 ms), $\mathrm{FOV}=440\times 399\;mm^{2}$, matrix=128×116, five slices (thickness = 10 mm, gap = 5 mm), no fat suppression, no acceleration, bipolar gradient, a bandwidth (BW) = 1560 Hz/pixel. These 2D multi‐slice data were acquired sequentially within a single 8.7 s breath‐hold. $T_{2}^{*}$ and PDFF maps were calculated for each volunteer using a magnitude‐only approach.^34^

Inline SASHA^20^ $T_{1}$ maps were acquired within a single 11 s breath‐hold with a balanced SSFP (bSSFP) readout using a work in progress protocol (WIP1041B) and the following imaging parameters: $\mathrm{FOV}=360\times 270\;mm^{2}$, matrix=128×96, single slice, 8 mm slice thickness, TR/TE = 2.77/1.39 ms, nominal readout FA of 45°, no fat suppression, no acceleration, and BW = 1085 Hz/pixel. SASHA $T_{1}$ maps were not acquired for the first two volunteers in runs 1 and 2.

MOLLI $T_{1}$ maps were acquired with a LiverMultiScan (Perspectum Ltd., Oxford, UK) protocol with the following imaging parameters: $\mathrm{FOV}=440\times 330\;mm^{2}$, matrix=192×144 (interpolated to 384×288), single slice, 8 mm slice thickness, TR/TE = 2.6/1.05 ms, no fat suppression, nominal readout FA of 35°, BW = 965 Hz/pixel, and GRAPPA with two‐fold acceleration in the PE direction; the acquisition schema was 5(1)1(1)1 indicating the number of images and pauses in the acquisition. Seven TIs were collected with three non‐selective adiabatic IR RF pulses played out before the first, sixth, and seventh single‐shot bSSFP readouts. The readout scheme was synchronized with the volunteer's R‐wave from their electrocardiogram (ECG) as MOLLI is a cardiac pulse sequence. However, for liver $T_{1}$ mapping a simulated ECG with an appropriate (˜1 s) RR interval can be used instead. The first of five samples started with a minimum TI of 100 ms and the subsequent four TIs were separated by the volunteer's RR interval (100, 100 + RR, 100 + 2RR, 100 + 3RR, 100 + 4RR); the last two samples were acquired at TIs of 180 and 260 ms. Data were acquired within a single nine heart‐beat long breath‐hold. Complex data from the first five TIs were fit to an IR model with a Look‐Locker correction to obtain an off‐line MOLLI $T_{1}$ map. Both SASHA and MOLLI $T_{1}$ maps were synchronized to the signal from a pulse oximeter and acquired with a bSSFP readout in a single slice at the liver's mid‐section.

For 3D $T_{1}$ mapping, the $T_{1}$ contrast of the liver tissue was obtained through a 3D VFA SPGR sequence with Dixon^35^ fat/water separation. The water‐only images were used for the calculation of the $T_{1}$ maps. Acquisition parameters were: $\mathrm{FOV}=450\times 366\times 3\;mm^{3}$, matrix=320×260×48, TR/TEs = 4.1/[1.23, 2.46] ms, BW = 1040 Hz/pixel. Data were acquired at nominal FAs of 2°, 2°, 15°, 15°,^36^ each during a 15 s end‐expiratory breath‐hold. CAIPIRINHA^37^ was used with an acceleration factor of 3 along the slice direction with 24 separate GRE reference lines. Spatial saturation was turned off as it perturbed the theoretical steady‐state signal.

A 2D multi‐slice GRE EPI sequence was used for $B_{1}^{+}$ mapping with fat saturation and nominal FAs of 65° and 130°.^36^ Acquisition parameters were: FOV=450×366 $mm^{2}$, matrix=64×52, slice thickness/spacing 8/2 mm, 15 slices interleaved, TR/TE = 10 000/11 ms without acceleration. The BW was 3906 Hz/pixel to achieve a minimum echo spacing of 0.3 ms, and the slices were acquired in an interleaved scheme. Each FA was acquired during a 10 s breath‐hold. This acquisition was used to calculate a $B_{1}^{+}$ map including corrections for slice profile effects and $B_{0}$‐gradient through‐slice variations.^38^

A 2D multi‐slice double echo spoiled GRE acquisition with magnitude‐ and phase‐reconstructed data was performed to compute a $B_{0}$ map. The $B_{0}$ map was used for distortion correction of the GRE‐EPI images through *fsl fugue* ^39^, ^40^ and modeling of $B_{0}$‐variations through slice in the $B_{1}^{+}$ map calculation. Acquisition parameters were TR/TE = 20/[4.78, 7.17] ms, $\mathrm{FA}=15^{{}^{\circ}}$, $\mathrm{FOV}=450\times 380\;mm^{2}$, matrix=64×54, slice thickness/spacing 8/2 mm, 15 slices, monopolar readout gradients, BW = 630 Hz/pixel, GRAPPA^41^ two‐fold acceleration in the PE direction, acquisition time 8.6 s breath‐hold.

The slice profile and $B_{0}$ gradient through slice corrected $B_{1}^{+}$ map was interpolated to the SPGR spatial resolution and multiplied by the nominal SPGR FAs to obtain the true excitation FAs. A correction for incomplete spoiling was applied to the water SPGR signal, through extended phase graph simulations,^42^ which was then fitted to the steady‐state SPGR signal model through a non‐linear least squares (NLLS) regression. The code to generate the $B_{1}^{+}$ and water $T_{1}$ maps is available on Github: https://github.com/gabrielaBelsley/3D‐T1‐Mapping‐Siemens.

Three circular regions of interest (ROIs) on each slice, each with a radius of four pixels, were used for the $T_{1}$ maps statistical analysis. To avoid potential biases in selecting the ROIs on the $T_{1}$ maps, the locations of the ROIs were initially drawn in the SPGR image acquired at a nominal $\mathrm{FA}=15^{{}^{\circ}}$ in vessel and bile free areas, avoiding the edges of the liver (Figure 3). Slices where three ROIs could not be easily fit within the central region without including bile or blood were excluded. Given this constraint together with a FOV that covers 14.4 cm of the liver in the slice direction, the majority but not the entire liver was included in the $T_{1}$ map calculation and statistical analysis. Three ROIs were also drawn on MOLLI and SASHA $T_{1}$ maps. Given the SASHA pixel size is almost double the VFA pixel spacing, an ROI size of two pixels radius was used for SASHA. For MOLLI, an ROI size of five pixels radius was used, reflecting the lower pixel spacing of ˜1.15 mm.

### 2.3 Statistical analysis

The estimated $T_{1}$ for each subject was calculated as a weighted mean (Eq. 1) and standard deviation (SD) (Eq. 2) of the $T_{1}s$ extracted from each ROI selected in each slice. The weights for each ROI were defined by the inverse of the standard error in the mean squared.

(1) $${w\mu }_{T_{1_{\text{Subject}}}}=\frac{\sum_{s=1}^{N_{\text{Slices}}}\sum_{r=1}^{N_{\text{ROIs}}}\sum_{p=1}^{N_{\text{Pixels}}}w_{s,r}T_{1_{s,r,p}}}{\sum_{s=1}^{N_{\text{Slices}}}\sum_{r=1}^{N_{\text{ROIs}}}N_{\text{Pixels}}w_{s,r}}$$

(2) $${w\sigma }_{T_{1_{\text{Subject}}}}=\sqrt{\frac{\sum_{s=1}^{N_{\text{Slices}}}\sum_{r=1}^{N_{\text{ROIs}}}\sum_{p=1}^{N_{\text{Pixels}}}w_{s,r}{\left(T_{1_{s,r,p}}-w\mu_{T_{1_{Subject}}}\right)}^{2}}{\sum_{s=1}^{N_{\text{Slices}}}\sum_{r=1}^{N_{\text{ROIs}}}N_{\text{Pixels}}w_{s,r}}},$$

where $w_{s,r}=\frac{N_{{\text{Pixels}}_{s,r}}}{\sigma_{s,r}^{2}}$. The sum over p runs over the N pixels in a given ROI, the sum over r runs over the different ROIs in a given slice and the sum over s runs over the slices in a given acquisition. $N_{\text{slices}}$ is the total number of slices, $N_{\text{ROIs}}$ is the total number of ROIs chosen per slice, $N_{\text{Pixels}}$ is the total number of pixels per ROI and $T_{1_{s,r,p}}$ is the $T_{1}$ value per pixel, in each ROI and corresponding slice.

#### 2.3.1 Evaluating inaccuracy

In the absence of a GS $T_{1}$ method in vivo, systematic relative differences between the measured weighted VFA $T_{1}$ (${w\mu }_{T_{1_{v,u}}}$) and the other two $T_{1}$ mapping methods (SASHA and MOLLI) were calculated using Eq. (3). MOLLI and SASHA $T_{1}$ values, $T_{1,{\text{reference}}_{v,u}}$ in Eq. 3, corresponded to weighted mean $T_{1}$ values from the three ROIs defined within the single acquired slice.

(3) $$\delta_{\text{inaccuracy}}=\frac{1}{N_{\text{volunteers}}\times N_{\text{Runs}}}\sum_{v=1}^{N_{\text{volunteers}}}\sum_{u=1}^{N_{\text{Runs}}}\frac{{w\mu }_{T_{1_{v,u}}}-T_{1,{\text{reference}}_{v,u}}}{T_{1,{\text{reference}}_{v,u}}}$$

As MOLLI is known to underestimate $T_{1}$ ^43^ and to be affected by fat,^24^ to calculate the VFA $T_{1}$ inaccuracy using the MOLLI $T_{1}$ maps as the reference $T_{1}$, it was necessary to forward simulate the VFA water $T_{1}$ into a MOLLI $T_{1}$ using a Bloch equation simulation.^44^ Three VFA slices, each of 3 mm thickness, fit within a single‐slice MOLLI acquisition of 8 mm thickness. Consequently, the mean VFA water $T_{1}$ from the nine ROIs drawn on the three VFA slices matching the MOLLI slice location were used as the $T_{1}$ input to the Bloch simulator. Additional inputs consisting of the $B_{0}$ value for each ROI, $T_{2}^{*}$, and PDFF were, respectively, used to correct the MOLLI simulated signal for off‐resonance, iron,^23^ and fat^24^ effects. The signal was also corrected for magnetization transfer effects.^21^ Other inputs included MOLLI sequence‐specific components such as the exact inversion RF pulse, gradient waveform, and the bSSFP readout pulse. The simulated transverse signal was fitted to an IR curve. The output was a Look‐Locker corrected simulated MOLLI $T_{1}$ using Deichmann‐Haase correction,^45^ which was compared to the acquired MOLLI $T_{1}$ data.

#### 2.3.2 Quantifying $B_{1}^{+}$ factor induced bias in the $T_{1}$ maps

$B_{1}^{+}$ factor bias in the $T_{1}$ maps was a source of systematic error we strived to minimize through a $B_{1}^{+}$ map acquisition. The residual correlation between $T_{1}$ and the $B_{1}^{+}$ factor, after applying the $B_{1}^{+}$ correction to the VFA, was computed for all mean $T_{1}s$ and $B_{1}^{+}$ factors within each ROI for each run. The correlation coefficient follows a t‐distribution with n‐2 degrees of freedom.^46^ The null hypothesis that there is no relationship between $T_{1}$ and $B_{1}^{+}$ factor was tested through a t‐test statistic at a significance level of 0.05. The slope of the linear regression between $T_{1}$ and $B_{1}^{+}$ factor indicated the strength of the relationship between the two variables (Eq. 4). A $B_{1}^{+}$‐related $T_{1}$ variation ($\Delta T_{1_{B_{1}^{+}}}$) is given by the product of the slope (b), from the linear fit between $T_{1}$ and $B_{1}^{+}$ factor, and the range of $B_{1}^{+}$ factor values ($\Delta B_{1}^{+}$) observed across the liver (Eq. 5).

(4) $$\mu_{T_{1}}=a+b\mu_{B_{1}^{+}}$$

(5) $$\Delta T_{1_{B_{1}^{+}}}=b\Delta B_{1}^{+}$$

The 95% confidence interval (CI) in the $B_{1}^{+}$‐related $T_{1}$ variation (Eq. 6) was calculated using a *t*‐test statistic with two degrees of freedom at a significance level of 0.05.^47^

(6) $$b\Delta B_{1}^{+}\pm t_{\left(\frac{0.05}{2},N_{\text{ROIs}}-2\right)}\times \Delta B_{1}^{+}\times \sqrt{\frac{s^{2}}{\sum_{i=1}^{N_{\text{slices}}}\sum_{j=1}^{N_{\text{ROIs}}}{\left(\mu_{{B_{1}^{+}}_{i,j}}‐\mu_{{B_{1}^{+}}_{\mathrm{Run}}}\right)}^{2}}},$$

where $s^{2}$ is the residual variance of the linear fit between the mean $T_{1}$ and mean $B_{1}^{+}$ factor.

#### 2.3.3 Evaluating repeatability

The method's precision in measuring $T_{1}$ was calculated from a one‐way analysis of variance (ANOVA) of the mean $T_{1}s$ from run 1 and run 2 across the 10 subjects, where the groups are the subjects. The within‐subject variance ($\sigma_{\text{within‐subject}}^{2}$ ) represents the measurement error, assuming there is no physiological variation in $T_{1}$ between the two measurements. The measurement error is equal to the residual mean square error in an ANOVA table. The repeatability coefficient (RC), which represents the measurement precision under a set of identical conditions,^16^ is 1.96 SDs of the measurement error times square root of 2 as we have two measurements for each subject (Eq. 7)^48^:

(7) $$\mathrm{RC}=1.96\sqrt{2}\sigma_{\text{within‐subject}}$$

To assess intra‐session repeatability, the difference in the weighted mean of run 1 and run 2 was analyzed. We took the null hypothesis to be that the mean difference between the weighted mean $T_{1}s$ ($\Delta \mu_{T_{1}}$) of the two runs was equal to zero. The 95% CI for the mean difference between the weighted mean $T_{1}s$ of the two runs is given by Eq. 8, ^48^:

(8) $$\Delta \mu_{T_{1}}\pm t_{\left(0.95,N_{\text{subjects}}-1\right)}\frac{\sigma_{\Delta \mu_{T_{1}}}}{\sqrt{N_{\text{subjects}}}}.$$

where $\Delta \mu_{T_{1}}={w\mu }_{T_{1_{\mathrm{Run}1}}}-{w\mu }_{T_{1_{\mathrm{Run}2}}}$ and the t‐statistic follows Student's *t*‐distribution with a significance level of 0.05 and 9 ($N_{\text{subjects}}-1$) degrees of freedom. The null hypothesis is rejected at the *α* = 0.05 level when the 95% CI does not contain zero.

Pearson's correlation coefficients between run 1 and run 2, and a linear regression slope close to 1 with a narrow 95% linear fit CI assessed the agreement in measured $T_{1}$ between the two runs. The same procedure was used to assess inter‐session repeatability between run 1 and run 3 as well as the repeatability of SASHA and MOLLI $T_{1}$.

#### 2.3.4 Evaluating $T_{1}$ homogeneity across the liver

In healthy volunteers, the $T_{1}$ values are expected to be homogeneous across the liver parenchymal tissue. The homogeneity of the measured $T_{1}$ values across the liver was assessed by calculating the dispersion of the weighted mean $T_{1}$ values at an ROI level through Eq. 9. In this equation, the inner sum runs over the different ROIs in a given slice, and the sum over s runs over the slices in a given acquisition. $N_{\text{Slices}}$ is the total number of slices, and $N_{\text{ROIs}}$ is the total number of ROIs chosen per slice.

(9) $${\left({w\sigma }_{T_{1}}\right)}_{\mathrm{ROI}}=\sqrt{\frac{\sum_{s=1}^{N_{\text{Slices}}}\sum_{r=1}^{N_{\text{ROIs}}}w_{s,r}{\left(\mu_{T_{1_{s,r}}}-{w\mu }_{T_{1_{\text{Subject}}}}\right)}^{2}}{\sum_{s=1}^{N_{\text{Slices}}}\sum_{r=1}^{N_{\text{ROIs}}}w_{s,r}}},$$

where $w_{s,r}=\frac{N_{{\text{Pixels}}_{s,r}}}{\sigma_{s,r}^{2}}$.

## 3 RESULTS

### 3.1 Phantom scans

**FIGURE 2:**
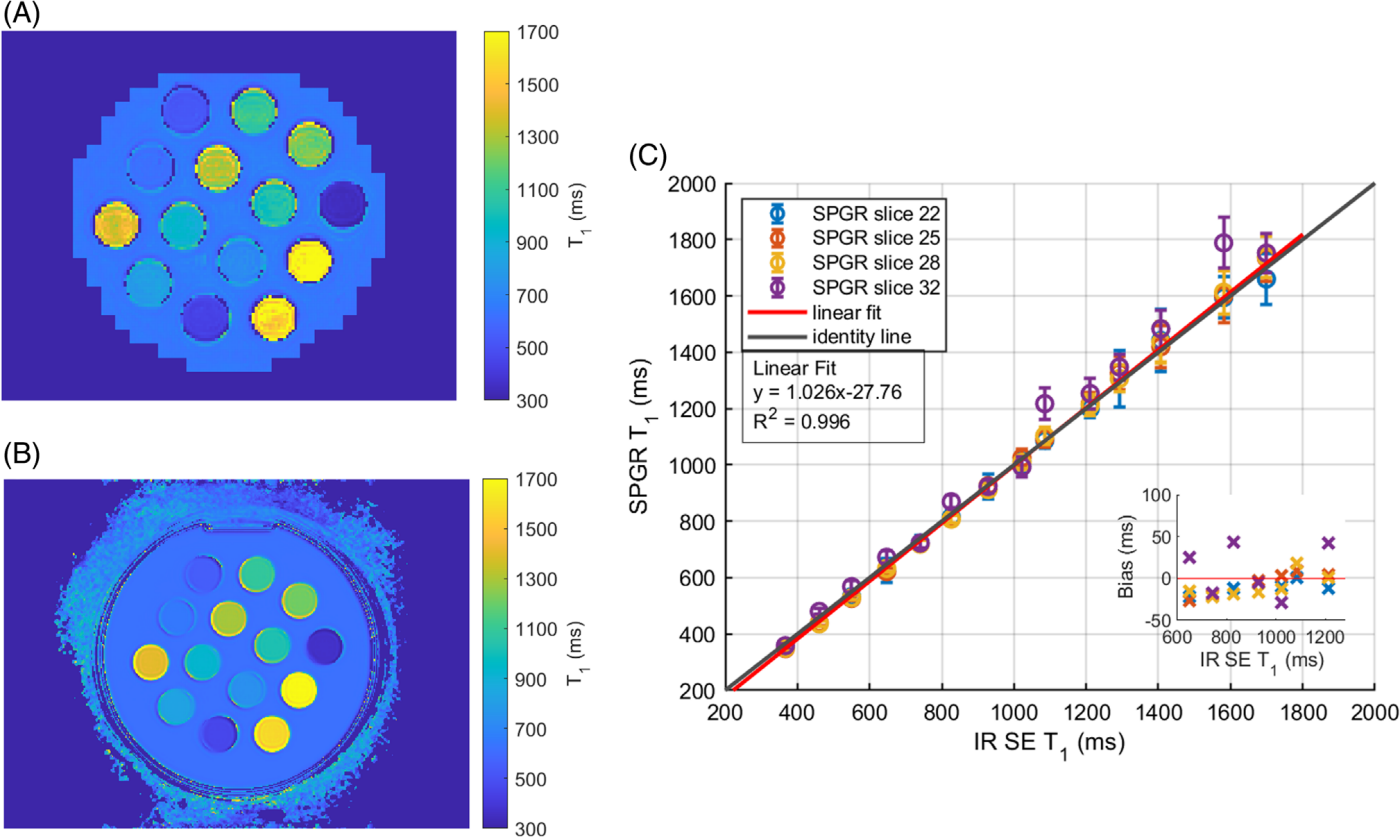
(A) 3D VFA SPGR $T_{1}$ map. (B) GS IR SE $T_{1}$ map. (C) Accuracy of the 3D $T_{1}$ VFA SPGR method on the phantom, for 14 different vials across four slices, measured against the IR SE. Linear fit, in red, resulted in a slope of 1.026 with an intercept of −27.76 ms. The $R^{2}$ was 0.996.

The $T_{1}$ map in the phantom showed an excellent agreement with the GS IR SE (Figure 2). The weighted least squares linear fit between the VFA SPGR $T_{1}$ and the GS IR SE resulted in a slope of 1.026 with a 95% CI [1.008, 1.043]. Across the physiologically relevant liver $T_{1}$ values at 3T (600–1300 ms), the interquartile range (IQR) relative $T_{1}$ error was [−2.3, 1.1]%. The weighted root mean squared normalized error between the SPGR $T_{1}$ and the GS $T_{1}$ was 2.8%. The weights were equal to the inverse of the SPGR $T_{1}$ SD squared.

### 3.2 In vivo scans

**FIGURE 3:**
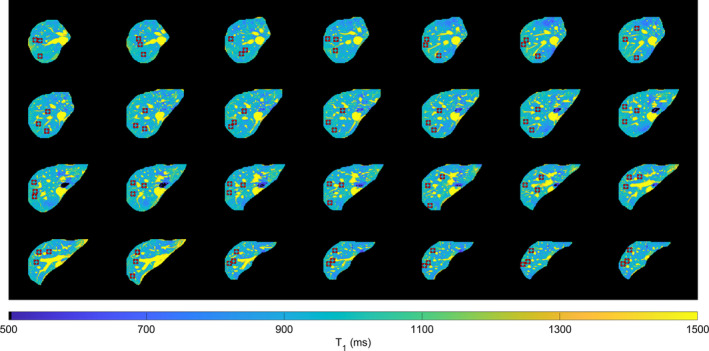
Representative VFA $T_{1}$ maps from a single volunteer overlaid with ROIs used in the statistical analysis (in red). Colormap range 500 ms to 1500 ms.

**FIGURE 4:**
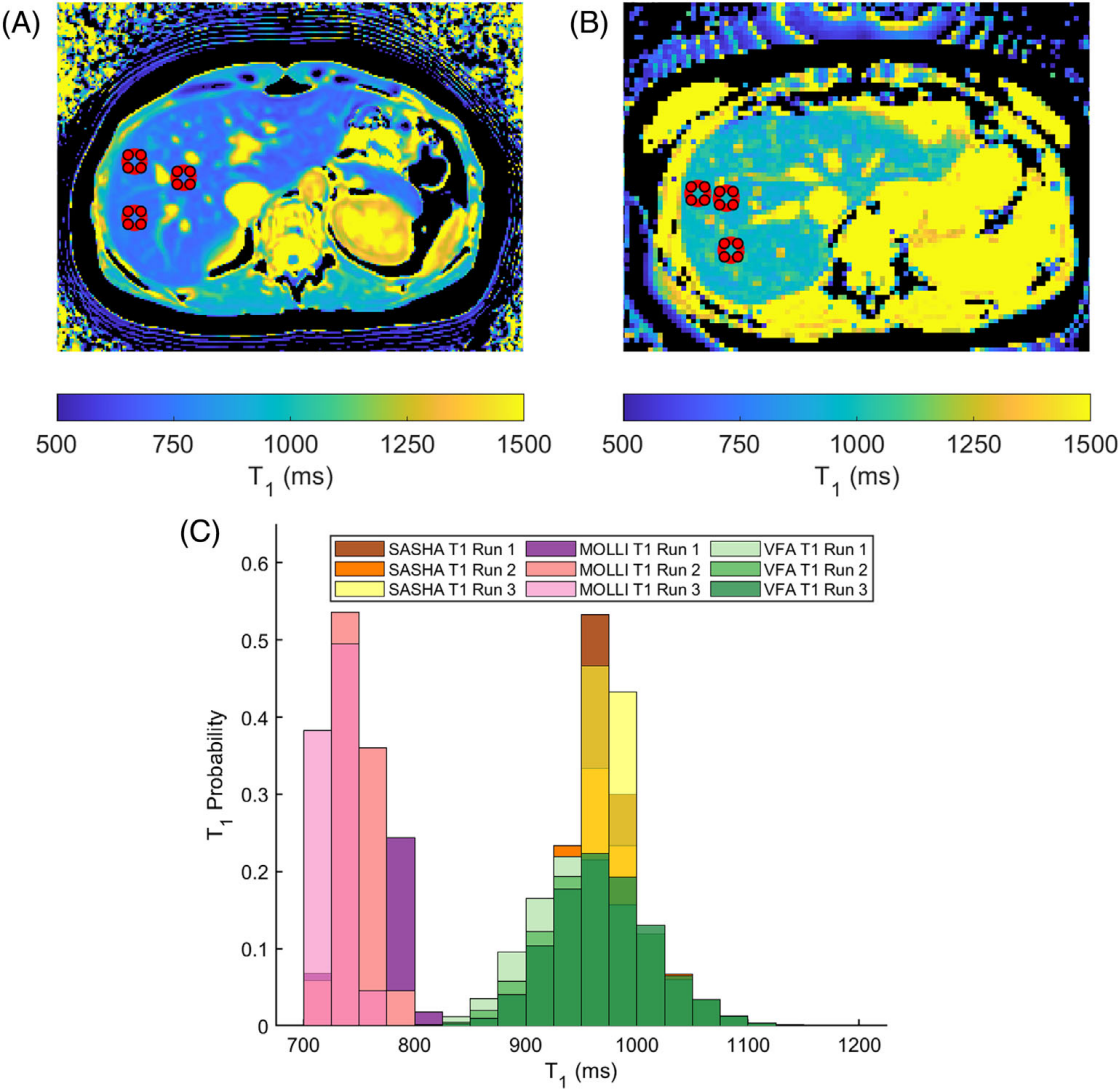
(A) MOLLI and (B) SASHA $T_{1}$ maps for the same volunteer as shown in Figure 3. (C) Histograms of $T_{1}s$ extracted from all the ROIs drawn for the three runs of the same volunteer. There are three different $T_{1}$ methods: Single slice MOLLI represented by the pink color scheme demonstrating $T_{1}$ underestimation; VFA $B_{1}^{+}$ corrected $T_{1}$ for runs 1/2/3 represented by the green color scheme and single slice SASHA $T_{1}s$ represented by the warm color scheme. 3D VFA $B_{1}^{+}$ corrected $T_{1}s$ have broader histogram widths compared to MOLLI or SASHA, but the three runs overlap showing high repeatability. The VFA $B_{1}^{+}$ corrected $T_{1}$ histogram modes agree well with SASHA.

The 3D VFA $T_{1},B_{1}^{+}$ corrected, maps from a single volunteer are shown in Figure 3, with the ROIs marked in red. Single slice MOLLI and SASHA $T_{1}$ maps for the same acquisition are shown in Figure 4 as well as the respective histograms of $T_{1}$ values.

**FIGURE 5:**
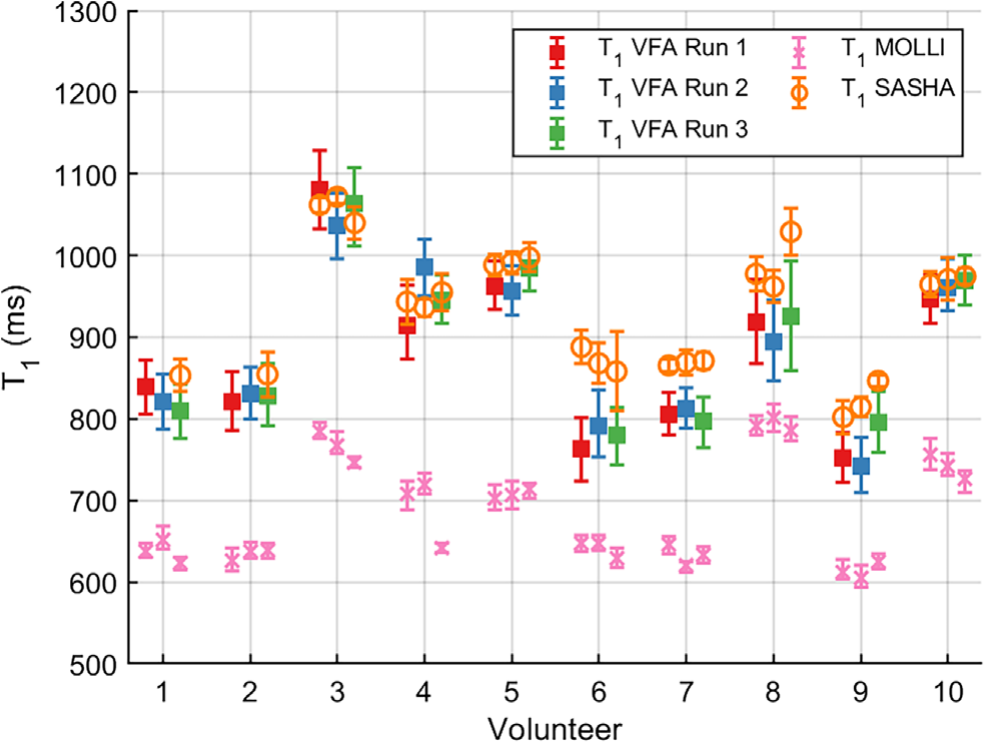
Median and IQR of VFA $T_{1}$ in ms, corrected for $B_{1}^{+}$ inhomogeneities, for the three runs acquired for each volunteer. Run 1 (red) and run 2 (blue) are a scan‐rescan, run 3 (green) is a second visit approximately 1 week later. Alternative $T_{1}$ methods: MOLLI (pink crosses) and SASHA (orange circles) are plotted as median ± IQR. SASHA $T_{1}$ maps were not acquired for the first two volunteers in runs 1 and 2.

Figure 5 compares the VFA $T_{1}$ $B_{1}^{+}$ corrected for the three runs acquired for each of the 10 volunteers. The three runs agreed well overall; the differences between the three runs were below 31 ms for 7 out of the 10 volunteers (higher differences observed for volunteers 3, 4, and 9).

VFA $T_{1}$ for all the ROIs across the 10 volunteers was on average 5% below SASHA and forward‐simulated MOLLI. For the VFA slices matching the SASHA and MOLLI slice location, the mean VFA $T_{1}$ was on average 4% below SASHA and 5% below forward‐simulated MOLLI $T_{1}$ values. The good agreement between liver VFA inaccuracies using all ROIs and inaccuracies for VFA ROIs matching the SASHA or MOLLI slice locations is an indication of $T_{1}$ homogeneity across the liver volume. Table 1 shows the mean $T_{1}$ values obtained for each volunteer measured with the three different $T_{1}$ methods as well as the PDFF and $T_{2}^{*}$ values.

**TABLE 1:** $T_{1}$ values averaged across all runs, slices, and ROIs for each volunteer using the 3D VFA $T_{1},B_{1}^{+}$ corrected, method proposed, SASHA and MOLLI $T_{1}$ mapping methods from a single slice.

Volunteer	3D VFA $T_{1}$ (ms)	$\text{SASHA}\;T_{1}$ (ms)	MOLLI $T_{1}$ (ms)	VFA forward simulated MOLLI $T_{1}$ (ms)	PDFF (%)	T2* (ms)
1	822	‐	645	627	1.5	14.2
2	824	‐	656	579	1.4	13.5
3	1053	1056	819	775	2.7	9.6
4	948	948	693	633	0.8	16.9
5	968	990	728	755	1.6	16.8
6	777	869	619	594	5.1	10.3
7	804	869	624	614	2.3	13.1
8	912	990	758	647	1.4	17.6
9	763	820	591	560	3.0	10.6
10	960	970	753	770	1.1	19.8

*Note*: Columns 6 and 7 show PDFF and $T_{2}^{*}$ values used respectively for the fat and iron correction in the VFA $T_{1}$ forward simulations into a MOLLI $T_{1}$ (column 5). SASHA $T_{1}$ maps were not acquired for the first two volunteers in runs 1 and 2.

**FIGURE 6:**
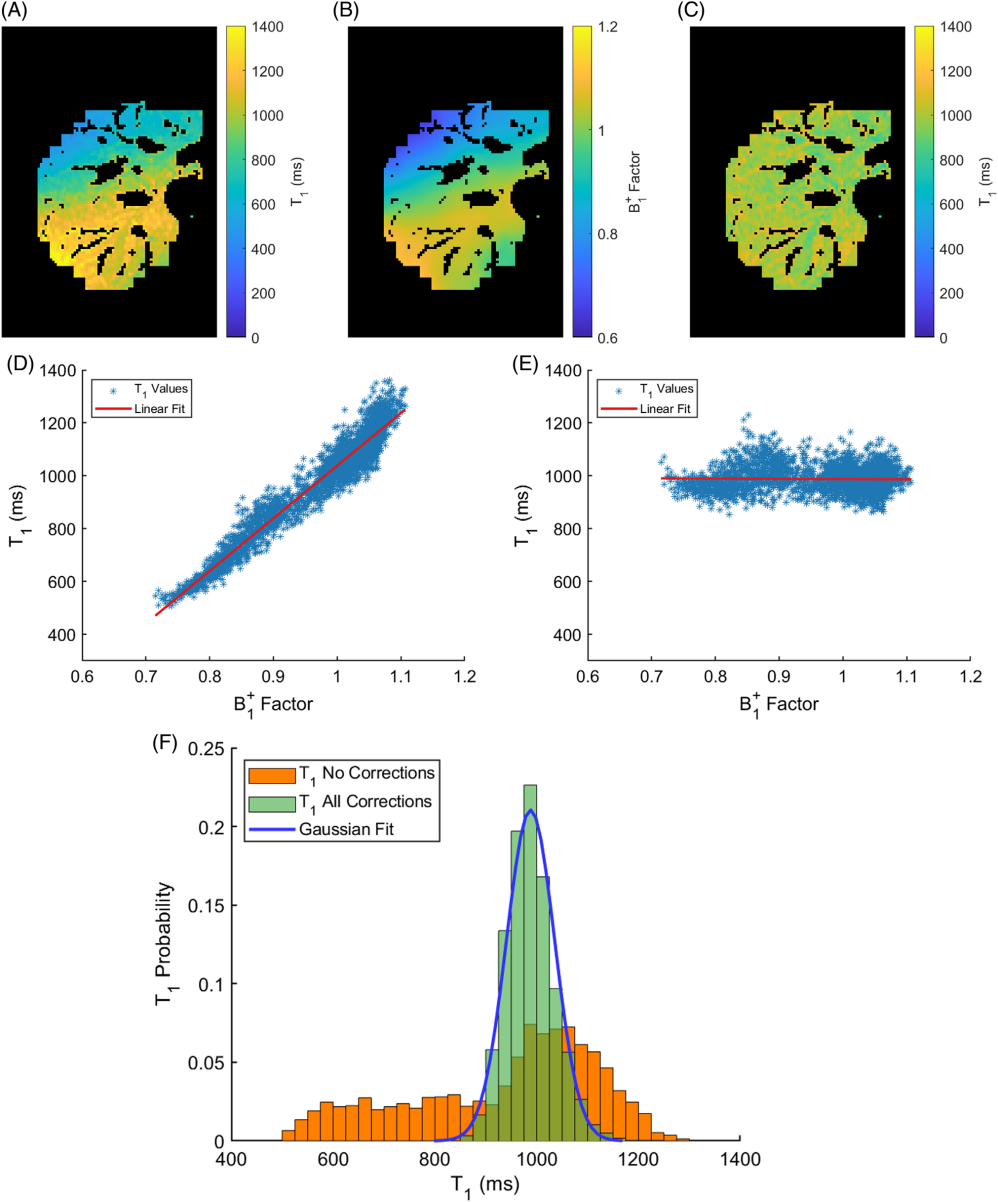
(A) Example in vivo $T_{1}$ map uncorrected for $B_{1}^{+}$ inhomogeneities, showing strong variations in $T_{1}$ especially along the vertical direction. (B) $B_{1}^{+}$ map used to correct the nominal FAs in the SPGR fit, resulting in a more homogenous (C) $B_{1}^{+}$ corrected $T_{1}$ map. Correlation between in vivo $T_{1}$ and $B_{1}^{+}$ values for (D) an uncorrected $T_{1}$ map and a (E) $T_{1}$ map corrected for $B_{1}^{+}$ inhomogeneities. Every point in the plots corresponds to a pixel from the ROIs drawn across the liver tissue of one volunteer. The $B_{1}^{+}$‐related variation in $T_{1}$ was (D) 781 ms with a 95% CI [774, 788] ms and in (E) it was reduced to −3 ms, with a 95% CI [−10, 4] ms containing zero. (F) $T_{1}$ distribution without any corrections (orange) and with the (green) $B_{1}^{+}$ correction as well as the incomplete spoiling correction.

One source of systematic errors in $T_{1}$ are errors in determining $B_{1}^{+}$ inhomogeneities. Figure 6A,B,D shows that there is strong correlation between $B_{1}^{+}$ and $T_{1}$ values for an in vivo VFA SPGR $T_{1}$ map uncorrected for $B_{1}^{+}$ inhomogeneities. However, after applying an accurate $B_{1}^{+}$ map to the SPGR FAs, the $B_{1}^{+}$‐related variation in $T_{1}$ was reduced from 781 ms with a 95% CI [774, 788] ms to −3 ms with a 95% CI [−10, 4] ms (Figure 6 C,E). Figure 6 (F) emphasizes the importance of correcting for $B_{1}^{+}$ inhomogeneities resulting in a much narrower $T_{1}$ distribution, approximating a normal distribution with a mean of 988 ms and a SD of 47 ms.

**FIGURE 7:**
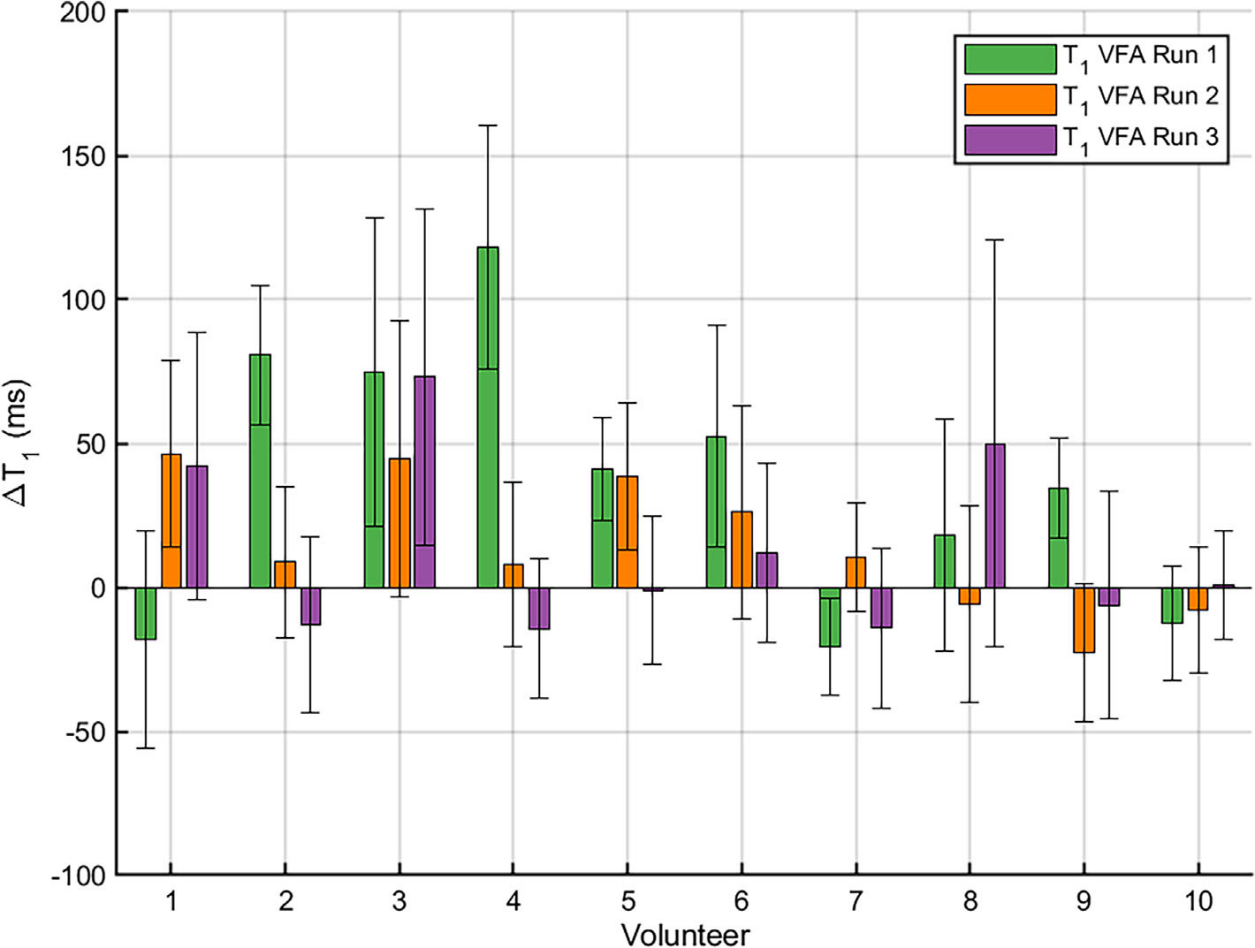
The range of $T_{1}$ values across the liver, for each subject and run, due to the measured $B_{1}^{+}$ factor variation, extrapolated from a linear fit between $T_{1}$ and $B_{1}^{+}$ (Eqs. 4 and 5). The slope of the linear fit between the mean $T_{1}$ and the mean $B_{1}^{+}$ factor in each ROI times the range of $B_{1}^{+}$ factors across the liver gives the $B_{1}^{+}$‐related $T_{1}$ variation (Eq. 5) plotted as bars for run 1 (green), run 2 (orange), and run 3 (purple) for each volunteer. The error bars represent the 95% CI of the slope, using a t‐test with 0.05 significance level, times the $B_{1}^{+}$ factor range (Eq. 6).

A linear fit between $T_{1}$ and $B_{1}^{+}$, according to Eq. 4, was carried out for each run and subject. In the ideal case, the slope should be close to zero as shown for the volunteer in Figure 6E. To assess the impact of any residual correlation between $T_{1}$ and $B_{1}^{+}$ on the $T_{1}$ maps, the slope was multiplied by the range of measured $B_{1}^{+}$ factors for each run and subject. The resulting spread of $T_{1}$ values is shown in Figure 7. The null hypothesis that there is no correlation between $T_{1}$ and $B_{1}^{+}$ factor was rejected for 10 out of 30 3D $T_{1}$ maps. These cases corresponded to a $B_{1}^{+}$‐related variation in $T_{1}$ larger than 34 ms, except for volunteer 7 run 1 (−21 ms). For the cases where the null hypothesis was not rejected, the absolute $B_{1}^{+}$‐related variation in $T_{1}$ was 17 ± 14 ms (μ±σ). A median $T_{1}$ SD of 31 ms was achieved at an ROI level, over all runs and volunteers, with an IQR of [27, 39] ms and extreme values of 20 and 62 ms.

**FIGURE 8:**
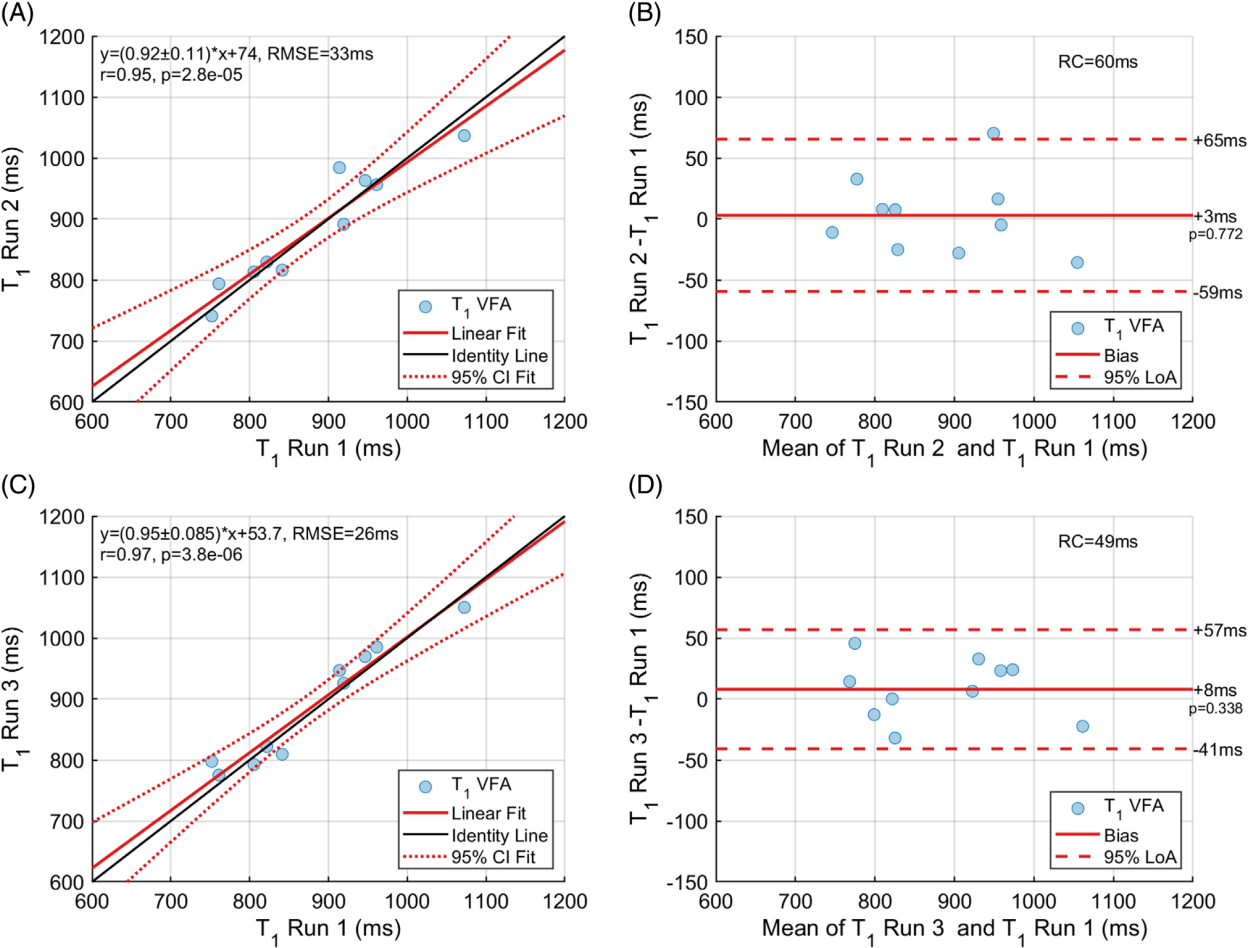
VFA $T_{1}$ intra‐session (A) correlation and (B) Bland Altman plots using the weighted mean $T_{1}$ for each subject. VFA $T_{1}$ inter‐session (C) correlation and (D) Bland Altman plots using the weighted mean $T_{1}$ (Eq. 1) for each subject.

Intra‐session and inter‐session (Figure 8) repeatability are illustrated using both correlation and Bland Altman plots. The VFA $T_{1}$ intra‐session RC was 62 ms and the inter‐session RC was 49 ms. Table 2 presents repeatability metrics for the alternative $T_{1}$ methods for comparison with the VFA $T_{1}$ method.

**TABLE 2:** Intra‐session and inter‐session repeatability metrics for 3D VFA $T_{1},B_{1}^{+}$ corrected, and alternative $T_{1}$ methods: MOLLI and SASHA.

$T_{1}$ method	Pearson correlation coefficient	Regression slope	Bias (ms)	95% Limits of agreement (ms)
VFA^a^	0.95	0.92 ± 0.11	+3	62
SASHA^a^	0.99	0.99 ± 0.05	−6	19
MOLLI^a^	0.98	0.97 ± 0.07	+1	26
VFA^b^	0.97	0.95 ± 0.09	+8	49
SASHA^b^	0.94	0.92 ± 0.14	+5	56
MOLLI^b^	0.93	0.83 ± 0.11	−14	49

*Note*: All correlation coefficients were statistically significant (*p* < 0.05). Correlation plot metrics include Pearson correlation coefficient and the regression slope with the associated standard error. Bland Altman plot metrics include bias and 95% limits of agreement.

^a^ Intra‐session: Run 1—Run 2.

^b^ Inter‐session: Run 1—Run 3.

## 4 DISCUSSION

Using an optimized image acquisition and processing pipeline for 3D $T_{1}$ mapping, a high accuracy was achieved characterized by a mean relative difference of −5% between VFA $B_{1}^{+}$ corrected $T_{1}$ and SASHA or forward‐simulated MOLLI $T_{1}$ in the livers of healthy volunteers. The liver $T_{1}$ variability at an ROI level was characterized by a median $T_{1}$ SD over all runs and volunteers of 31 ms, an IQR of [27, 39] ms and extreme values of 20 ms and 62 ms. The method showed a high repeatability, with a within‐subject $T_{1}$ SD of 21 ± 5 ms resulting in an intra‐session RC of 60 ms. The $T_{1}$ differences between scan and rescan had on average a + 3 ms bias (95% CI [−20, 26] ms, *p* = 0.772) with limits of agreement (LoA) between −59 ms and 65 ms and a correlation coefficient of 0.95. The $T_{1}$ difference between the two visits, approximately 1 week apart, had an average $T_{1}$ bias of +8 ms (95% CI [−10, 26] ms, *p* = 0.338) and a 95% LoA between −41 and 57 ms, with a RC of 49 ms. Neither the 3 or 8 ms average bias were statistically significant. Overall, the RC of the developed VFA $B_{1}^{+}$ corrected $T_{1}$ method lies between 50 and 60 ms.

The $T_{1}$ range of 763 to 1053 ms obtained in our study is within the range of liver $T_{1}s$ reported in the literature at 3T.^30^, ^49^ The biological variation in $T_{1}$ is expected and related to the age and sex of the volunteers. Pre‐menopausal women tend to have higher liver $T_{1}$ relaxation times compared to male or post‐menopausal women.^17^, ^30^ In our study, $T_{1}$ values between 947 and 1050 ms corresponded to female volunteers.

Very few studies have reported the accuracy of the VFA $T_{1}$ mapping. Preibisch et al.^50^ and Baudrexel et al.^51^ both used a multi‐slice IR‐EPI as the reference $T_{1}$, without breath‐holding as it was applied to the brain, and obtained an inaccuracy in white matter VFA $T_{1}$ of 6.5% and 4.5%, respectively. These mean absolute errors are comparable to those obtained in this work with SASHA and MOLLI. Stikov et al.^52^ demonstrated both in phantom and across 10 healthy volunteers an overestimation of VFA $T_{1}$ relative to IR or Look‐Locker IR. In vivo differences in $T_{1}$ larger than 30% were observed in white matter. The authors hypothesized that inaccurate $B_{1}^{+}$ mapping or incomplete spoiling may explain this overestimation. They called attention to the need for a “universal calibration scheme”, necessary to harmonize $T_{1}$ mapping across scanners and advise that $T_{1}$ mapping techniques should be validated against the GS IR. The fact that we obtained a high accuracy in the IR SE validation in the $T_{1}$ phantom with our optimized pipeline, and in vivo VFA $B_{1}^{+}$ corrected $T_{1}$ relative differences assessed with the forward‐simulated VFA MOLLI and SASHA were on average − 5%, confirms that we have been able to successfully correct for the main systematic errors affecting VFA $T_{1}$ mapping at a single field strength.

MOLLI $cT_{1}$ repeatability measurements reported in vivo across 22 healthy volunteers had a mean bias of −7.5 ms and 95% LoA [−53.6, 38.5] ms with a RC of 46.1 ms.^18^ Another MOLLI $cT_{1}$ repeatability study^53^ showed a mean bias of −13 ms and 95% LoA [−81, 56] ms. Our VFA $B_{1}^{+}$ corrected $T_{1}$ mapping approach achieved comparable LoA and RCs to the two MOLLI $cT_{1}$ studies, which is a single slice $T_{1}$ method.

Tadimalla et al.^28^ assessed the bias, repeatability, and reproducibility of VFA $T_{1}$ mapping in the liver at field strengths of 1.5 T and 3.0T across three major vendors. The authors report on average a 30% overestimation of $T_{1}$ with VFA methods, which is six times higher than the bias obtained in our study; Repeatability measures were more than three times larger than in our study (5% vs. 16%) and between‐subject variation were eight times worse in their study (7% vs. 56%). These results are likely due to the absence of $B_{1}^{+}$ correction to the VFA $T_{1}$ maps. The lack of a validated $B_{1}^{+}$ mapping method available across different scanner manufacturers was the reason behind its exclusion from their study, and is also emphasized as a concern by the QIBA DCE‐MRI Biomarker Committee.^54^ A robust, accurate, and precise $B_{1}^{+}$ mapping method^38^ was used in this study and can be easily adapted to other VFA $T_{1}$ mapping studies.

Larger inter‐session variations compared to intra‐session were expected. A closer look at these data suggest that one data point (outside of the LoA) had an intra‐session difference that was twice as large as the second largest difference. When eliminating this data point, the RC decreased from 60 to 43 ms. This volunteer reported difficulty in hearing the automatic breath‐hold instructions and holding their breath consistently at the same position for all scans. The method's sensitivity to large breath‐hold mismatches is one of its main disadvantages compared to MOLLI or SASHA where the $T_{1}$ map is acquired in a single breath‐hold, provided there is no breathing during the single breath‐hold.

In our study, we chose to use 4 FAs to achieve a precise measurement of $T_{1}.$ ^36^ Even though it is possible to calculate a $T_{1}$ map from just two FAs, it would have resulted in a within‐subject $T_{1}$ SD larger than 21 ms. In a previous study,^36^ we showed that an approximately 5% decrease in $T_{1}$ coefficient of variation was observed when increasing the number of FAs from two to four. The number of FAs chosen will be a compromise between $T_{1}$ precision and patient comfort. The minimum number of breath‐holds for 3D $T_{1}$ mapping would be four (two SPGR FAs and two $B_{1}^{+}$ FAs); the $B_{0}$ field map could be extracted from the dual‐echo SPGR acquisition. The larger number of breath‐holds will be more prone to anatomical misalignments between FAs; $T_{1}$ maps at the liver extremities will be more sensitive to misalignments. Some volunteers showed small misalignments in the breath‐holds between different FAs, but the method seems to be relatively robust to these mismatches given the within‐subject SD and RCs achieved. Registration should be carefully evaluated as it will move the anatomy to locations with different $B_{1}^{-}$ factors. The $B_{1}^{-}$ and the proton density are assumed to be constant on a pixel‐by‐pixel basis over the various acquisitions used to determine the $B_{1}^{+}$ factor or estimate $T_{1}$.

Systematic errors arising from residual $B_{1}^{+}$ inhomogeneities in the $T_{1}$ maps were statistically significant in one third of the cases. One reason for a correlation between $T_{1}$ and $B_{1}^{+}$ factor is an inaccurate determination of the $B_{0}$ values used to correct the through slice dephasing affecting the DAM ratio and, thus, the $B_{1}^{+}$ map.^38^ The edges of the liver or tissue boundaries, such as the bile ducts, portal vein, or gall bladder, are regions susceptible to large dephasing and thus phase wraps as well as low spin density, resulting in a large variance in the $B_{0}$ maps.

Our study covered most of the liver tissue across all volunteers, but not the entire liver. Nevertheless, even with a 14.4 cm coverage along the slice direction, there was very little liver tissue present on the outer slices and, consequently, there are more partial volume effects for these slices. Moreover, the dome of the liver or the edges are susceptible to large dephasing and thus phase wraps resulting in large variances in $B_{0}$ maps which are needed for the $B_{1}^{+}$ map calculation. Increasing the number of slices would increase the 15 s breath‐hold which may be challenging for patients. Increasing the slice thickness is an option and would increase the acquisition SNR. The downside of thicker slices is the inclusion of more blood vessels and bile ducts within the liver tissue voxel, biasing the liver $T_{1}$ measurement.

One underlying assumption in this study was that liver $T_{1}$ did not vary during the inter‐session repeatability scans. Limitations of our study include the number of volunteers and the absence of a GS $T_{1}$ mapping method in vivo. In addtion, the VFA $B_{1}^{+}$ corrected $T_{1}$ method does not compensate for the impact of iron. Thus, in patients with elevated iron, the measured $T_{1}$ will be biased compared to a $cT_{1}$ ^23^ method that reflects the state of the water in the liver tissue, although less strongly than MOLLI measurements due to the $T_{2}$ bias in the latter. Future work could comprise applying an iron correction of $T_{1}$ on the measured VFA $T_{1}$.

Another limitation of the two‐echo Dixon based VFA SPGR method for $T_{1}$ mapping is its inaccuracy in the presence of fat content in the liver. As shown in Figure S2 the $T_{1}$ inaccuracy increases as the fat percentage increases; this stems from the imperfect fat and water signal separation using the two‐echo Dixon method (Figure S4). Therefore, for accurately mapping water liver $T_{1}$ in cases of patients with fat percentages in the liver higher than 10%, the method would require using more than two echoes together with a correction for $T_{1}$‐weighting introduced by FAs near or above the Ernst angle. For fat fractions up to 10% the $T_{1}$ error is 3% (24 ms for a $T_{1}$ of 800 ms), which is comparable to the imprecision of the method measured in vivo (21 ± 5 ms). Despite this limitation, the proposed method can be used for patients with cirrhosis or hepatocellular carcinoma where the percentage of fat in the liver is usually low and should be adapted for patients with high PDFF in the liver.

This study demonstrated the feasibility of using widely available pulse sequences for an accurate and precise 3D liver $T_{1}$ mapping on a Siemens scanner. The next step toward developing a scanner‐agnostic 3D $T_{1}$ mapping, suitable for multi‐center and vendor $T_{1}$ comparisons, is to deploy the method using another vendor's scanner and test its reproducibility. For example, vendor differences in pulse sequences implementations such as spoiling, RF excitation profile, or DIXON fat‐water separation may yield differences in the calculated liver $T_{1}$, making it essential to explore these potential variations and evaluate their impact.

## 5 CONCLUSIONS

In conclusion, a method for 3D VFA $T_{1}$ mapping at 3T covering the majority of the liver was developed, using only widely available pulse sequences and seven breath‐holds, that is accurate, precise, and shows high intra‐ and inter‐session repeatability. The outlined method should increase the clinical availability of robust liver $T_{1}$ (and $B_{1}^{+}$) mapping methods. This method also provides a template for the deployment of $T_{1}$ mapping across different scanners and vendors, thus facilitating multicenter studies, essential in consortium research studies, as well as clinical trials of pharmaceutical products.

## FUNDING INFORMATION

This work was supported by funding from the Engineering and Physical Sciences Research Council (EPSRC), Medical Research Council (MRC) [grant number EP/L016052/1] and the NIHR Oxford Biomedical Research Centre.

## CONFLICT OF INTEREST STATEMENT

Gabriela Belsley is employed by Siemens Healthcare GmbH, and Matthew D. Robson is an employee and shareholder of Perspectum Ltd.

## Supporting information


**DATA S1:** Supporting Information.

## Data Availability

The code for the 3D VFA SPGR $T_{1}$ mapping is available here: https://github.com/gabrielaBelsley/3D‐T1‐Mapping‐Siemens.
