# Supplementary material for: Accurate and precise in vivo liver 3D T 1 mapping at 3T
Source: Magn Reson Med. 2025 Feb 4;93(6):2331–45. doi: 10.1002/mrm.30448 (PMC11971508; doi:10.1002/mrm.30448)
Supplement: Supplementary file 1 — DATA S1: Supporting Information. [file MRM-93-2331-s001.docx]

**Supporting Information**

$\boldsymbol{T}_{\boldsymbol{1}}$ **accuracy of the 2-echo Dixon VFA SPGR in a Fat Phantom**

We assessed the hypothesis of whether the 2-echo Dixon 3D variable flip angle (VFA) spoiled gradient recalled echo (SPGR) sequence provided an accurate fat-water separation for $T_{1}$ mapping. A Calimetrix phantom (Madison, WI, USA) containing 5 agar-based vials with increasing percentage of fat (5%, 10%, 20%, 30%, 40%) and vials with varying $T_{1}$ and no fat was scanned. The 5 fat vials (emulsions of peanut oil in agar gels doped with NiCl_2_) and a vial with a $T_{1}$of approximately 800 ms (agar gel doped with NiCl_2_) and no fat were evaluated.

An inversion recovery spin echo (IR SE) with fat saturation was used as the gold standard for mapping the water $T_{1}$in the fat vials. Acquisition parameters were: inversion times of 25 ms, 50 ms, 75 ms, 100 ms, 200 ms, 300 ms, 400 ms, 600 ms, 800 ms, 1200 ms, 1600 ms, 2300 ms, 3000 ms, 4000 ms, 6500 ms, TR/TE = 10 s/12 ms, FoV= 240×180 mm^2^, matrix=128×96, a single axial slice of 5 mm thickness, without acceleration, phase partial Fourier off, interpolation on. The BW was 130 Hz/Pixel. The average $T_{1}$of the fat vials were approximately equal across all the vials (Table S1), indicating fat saturation effectively suppressed the fat signal. Figure S1 shows the IR SE and the VFA $T_{1}$maps.


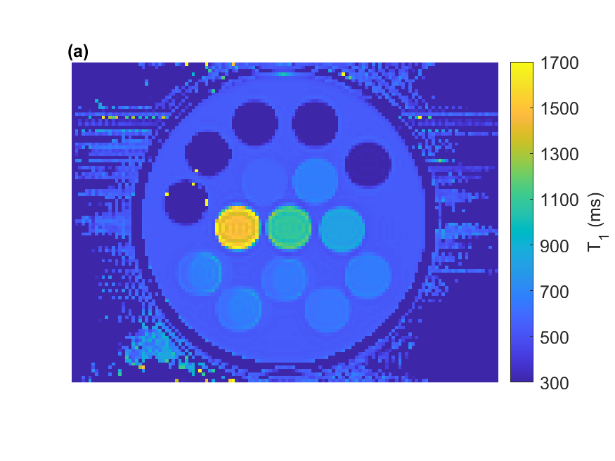


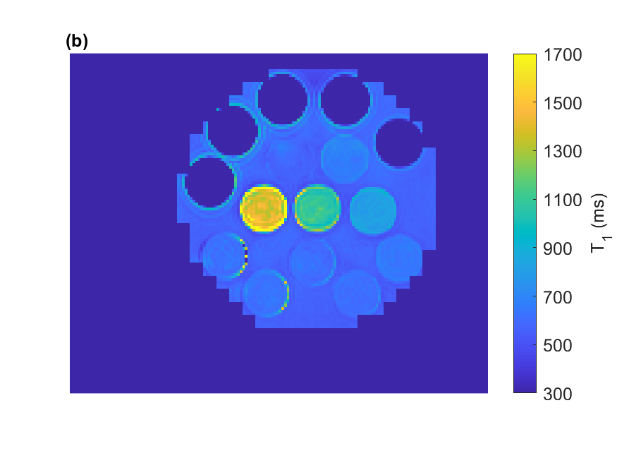


Figure S1: (a) Gold standard IR SE $T_{1}$ map and (b) VFA SPGR $T_{1}$ map. The lower 5 vials correspond to the fat vials with increasing percentage of fat from right to left. The step wise cut in the VFA SPGR $T_{1}$ map comes from using a mask during the $B_{1}^{+}$ and $T_{1}$ calculation.

Table S1 shows the average $T_{1}$and standard deviation calculated across the vials with different fat percentages for the VFA SPGR Dixon and the IR SE $T_{1}$method.

Table S1: $T_{1}$ values (average$\pm$ standard deviation) calculated by drawing ROIs with approximately 115 pixels for each vial with fat fractions of 0%, 5%, 10%, 20%, 30%, 40% for both the VFA SPGR Dixon proposed method and the gold standard IR SE $T_{1}$ method.

| Fat Percentage | $T_{1}$SPGR Dixon ($\mu\pm\sigma)$  (ms) | $T_{1}$IRSE ($\mu\pm\sigma)$  (ms) |
| --- | --- | --- |
| 0 % | 810 $\pm$ 17 | 815 $\pm$10 |
| 5 % | 644$\pm$ 14 | 656$\pm$ 5 |
| 10 % | 612$\pm$ 10 | 630$\pm$6 |
| 20 % | 618$\pm$ 20 | 645 $\pm$12 |
| 30 % | 628$\pm$ 15 | 661 $\pm$16 |
| 40 % | 626$\pm$ 23 | 660$\pm$23 |


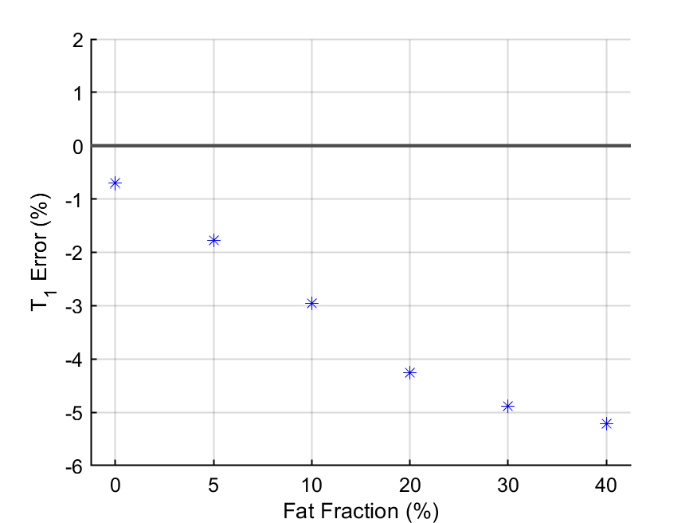
Figure S2 shows that the 2-echo Dixon VFA SPGR $T_{1}$ error increases as the fat fraction increases. The $T_{1}$ error was -0.7%, -1.8%, -3.0%, -4.3%, -4.9%, -5.2% for fat fractions of 0%, 5%, 10%, 20%, 30% and 40%, respectively.

Figure S2: $T_{1}$ Error in percentage, normalized by the $T_{1}$ gold standard IR SE. The $T_{1}$ error increases as the fat fraction increases.

To check if the $T_{1}$ error in Figure S2 was caused from an error in separating the fat and the water signal using the 2-echo Dixon, we compared the fat fraction calculated from a dual-echo and a multi-echo acquisition. The multi-echo consisted of a 6-echo SPGR acquisition with the following acquisition parameters: TR = 9 ms, TEs = [1.06, 2.20, 3.34, 4.48, 5.62, 6.76] ms, slice thickness 4 mm, FA = 4°, FoV = 400×330 mm^2^, matrix = 160×132, BW = 1040 Hz/pixel. The MAGO^1^ fat-water separation algorithm was used for the multiple echo Proton Density Fat Fraction (PDFF) calculation (Figure S3). For the dual-echo Dixon SPGR, the fat fraction was calculated from the ratio between the fat images and the sum of the fat and water images for a 3° excitation flip angle.


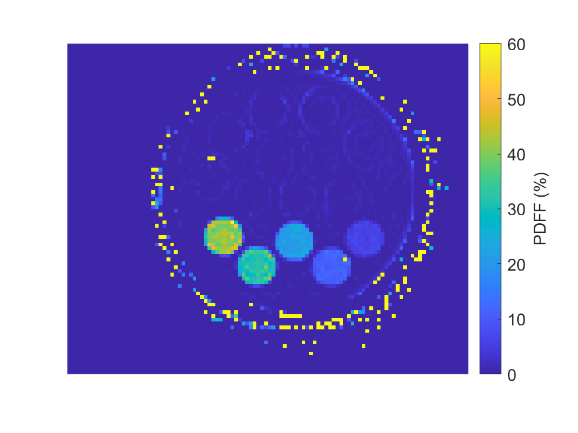


Figure S3: PDFF map from the multi-echo acquisition.

Figure S4 (a) shows that the 2-echo Dixon is overall accurate at estimating PDFF over a wide range of fat fractions using a low FA of 3°. However, it’s not accurate for higher FAs as shown in Figure S4 (b). As the FA value increases above 6 degrees, the PDFF error increases which subsequently results in an error in $T_{1}$ as shown in Figure S2. Table S2 shows the PDFF error using a dual-echo relative to the multi-echo method for different FA values. FAs above the Ernst angle introduce different $T_{1}$-weightings into the fat and water signal which results in fat fraction overestimation as showed in Kühn et al^2^.


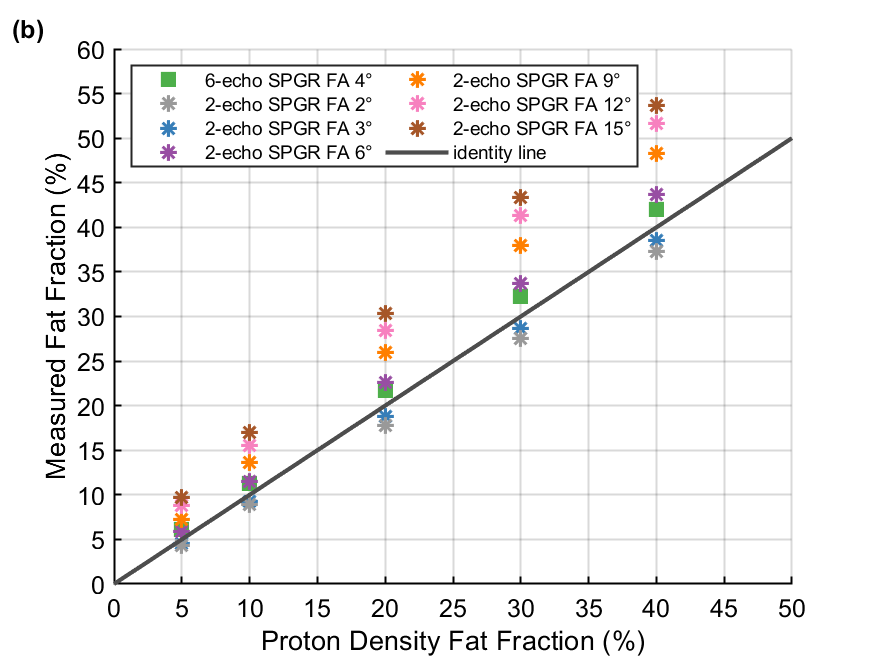

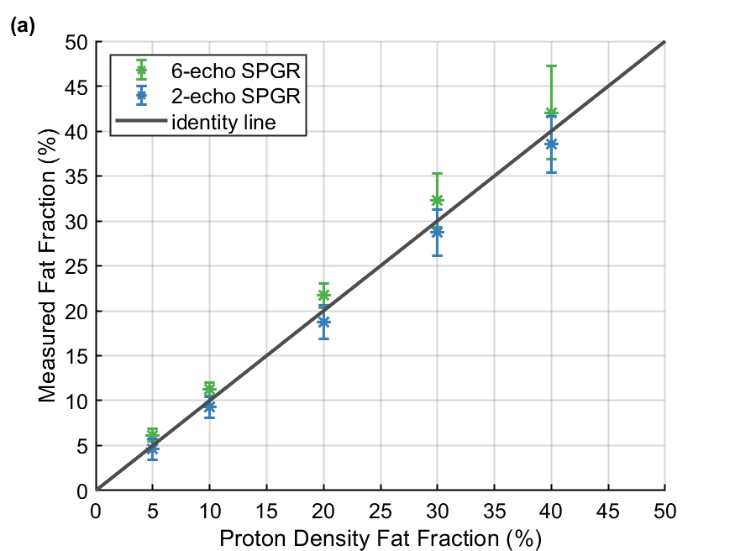


Figure S4: Measured percentage fat fraction as a function of percentage PDFF in the Calimetrix phantom factsheet for the (a) multi-echo acquisition using 6 echoes in green acquired at a FA of 4° and the dual-echo acquisition in blue acquired at a FA of 3°, and in (b) including also different dual-echo SPGR FAs with values of 2**°** (gray), 3° (blue), 6° (purple), 9° (orange), 12° (pink) and 15° (brown).

|  | PDFF 5% | PDFF 10% | PDFF 20% | PDFF 30% | PDFF 40% |
| --- | --- | --- | --- | --- | --- |
| FA 2**°** | -1.9 | -2.3 | -3.9 | -4.8 | -4.8 |
| FA 3**°** | -1.6 | -2.0 | -3.0 | -3.6 | -3.5 |
| **FA 6°** | -0.3 | 0.2 | 0.9 | 1.4 | 1.7 |
| FA 9**°** | 1.1 | 2.4 | 4.3 | 5.7 | 6.2 |
| FA 12**°** | 2.7 | 4.3 | 6.8 | 9.0 | 9.6 |
| FA 15**°** | 3.5 | 5.8 | 8.7 | 11.0 | 11.6 |

Table S2: Difference between the PDFF estimated from a 2-echo Dixon SPGR at FAs of 2**°**,3**°**,6**°**,9**°**,12**°** and 15**°** and the 6-echo SPGR at a FA of 4**°**.

$\boldsymbol{T}_{\boldsymbol{1}}$ **Map Validation using a Gold Standard** $\boldsymbol{T}_{\boldsymbol{1}}$ **Map**

A gold standard (GS) $T_{1}$ map using a slice-selective inversion-recovery spin echo (IR SE) was acquired on the $T_{1}$phantom using TIs of [25, 50, 75, 100, 200, 300, 400, 600, 800, 1200, 1600, 2300, 3000, 4000, 5000]ms, TR/TE=9s/12ms, FoV=$225\times300 mm^{2}$, matrix=$144\times192$, 6mm slice thickness, BW=130 Hz/pixel, Phase Partial Fourier Off, Interpolation On, no acceleration. The acquisition time for each TI was 21 minutes and 47 seconds.

To calculate the GS $T_{1}$ map (Figure S5), the signal in each pixel was fit using a three-parameter non-linear IR model:

$$S_{n}=a-be^{\left( \frac{-TI_{n}}{T_{1}} \right)} ,$$

where $S_{n}$ is the signal intensity, $a$ is the signal at $TI=\infty$, after its full recovery to the equilibrium longitudinal magnetisation $M_{z}$, and $a-b$ is the signal at $TI=0$. Initial guesses for $a$ is the signal from the largest TI (5000 ms) and for $b$ is two times the signal at the largest TI. $T_{1}$ was initialised to 500 ms. The subscript $n$ denotes the index of the TI; in this work the signal was measured at 15 different TIs. For each fit, the TI corresponding to the minimum signal intensity is determined and all the TIs less than or equal to the TI with the minimum signal intensity are assigned a negative value, i.e., samples which have not reached the null intensity. A second fit is carried out with the minimum signal intensity changed back to positive. The fit with the smallest sum squared errors value is chosen.

Figure S5. Inversion-recovery spin echo gold standard $T_{1}$ map of the $T_{1}$phantom with mean $T_{1}$s varying between 367 ms and 1699 ms.

$\boldsymbol{B}_{\boldsymbol{1}}^{\boldsymbol{+}}$ **Map Validation using a Gold Standard** $\boldsymbol{B}_{\boldsymbol{1}}^{\boldsymbol{+}}$ **Map**

A 3D non-selective GRE sequence gave a GS $B_{1}^{+}$ map using the DAM^3^. Two acquisitions at nominal FAs of 30˚ and 60˚ were acquired in an interleaved manner to avoid signal drift over time. Acquisition parameters were: FoV=$192\times192\times10 mm^{3}$, matrix=$48\times48\times16$, TR/TE=10s/2ms, BW=1000 Hz/pixel, Slice/Phase Partial Fourier Off, Interpolation On, no acceleration. The acquisition time for each FA was 4 hours and 16 minutes.

Figure S6 shows the $B_{1}^{+}$ GS maps in the $T_{1}$ phantom. Given the central slices in 3D acquisitions have a rectangular or ideal slice profile, the double angle method^1^ was used to calculate the true FAs ($\alpha$) exciting the spins in the phantom:

$$\alpha\left( \mathbf{r} \right)=arccos\left( \left| \frac{S_{2}\left( \mathbf{r} \right)}{2S_{1}\left( \mathbf{r} \right)} \right| \right)$$

It takes the ratio between two signals from fully relaxed spins, $S_{2}$ and $S_{1}$, acquired respectively at nominal FAs $2\alpha$ and $\alpha$, and **r**=(x,y,z).


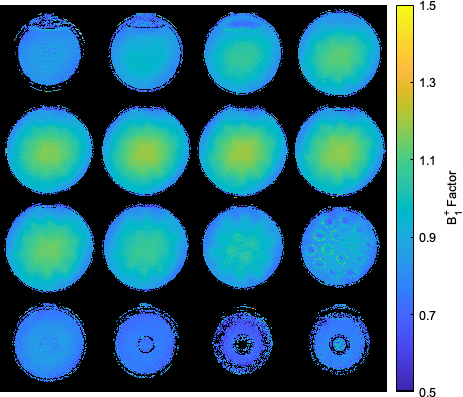


Figure S6. $B_{1}^{+}$ gold standard maps of the $T_{1}$phantom across 16 slices.

To check whether the 3D GRE DAM $B_{1}^{+}$ could be used as the gold standard $B_{1}^{+}$ mapping method in the $T_{1}$ phantom, the SPGR $T_{1}$ map with the FAs corrected by the 3D GRE DAM $B_{1}^{+}$ was compared to the IR SE $T_{1}$ map. A good agreement was achieved with the IR SE, as shown in Figure S7 with a slope close to the identity line.

Figure S7. Validation of the gold standard $B_{1}^{+}$ mapping method. (a) SPGR $T_{1}$ map with the SPGR FAs corrected by the 3D GRE $B_{1}^{+}$ map. The signal was corrected for incomplete spoiling. (b) Validation of the gold standard $B_{1}^{+}$ mapping method. Linear fit of the SPGR $T_{1}$ map to the gold standard IR SE. The slope from the linear fit was 1.03 with a 95% confidence interval between 1.019 and 1.046.

The temperature of the 3D SPGR $T_{1}$ mapping protocol and the GS $B_{1}^{+}$ and $T_{1}$ mapping was monitored overnight using fibre optic probes attached to the surface of the phantom. The mean ± standard deviation temperature was 20±0.4$℃$ over the duration of 19 hours. The magnitude of these temperature variations result in insignificant $T_{1}$ changes for NiCl_2_ filled vials. Stupic et al.^4^ measured $T_{1}$ variations of 1.3% in a NiCl_2_ array over a temperature range from 18$℃$ to 26$℃$.

References

1. Triay Bagur A, Hutton C,Irving B, Gyngell ML, Robson MD, Brady M.Magnitude‐intrinsic water–fat ambiguity can beresolved with multipeak fat modeling and a multipointsearch method. Magn Reson Med. 2019;82:460–475. https://doi.org/10.1002/mrm.27728

2. Kühn JP, Jahn C, Hernando D, et al. T1 bias in chemical shift-encoded liver fat-fraction: role of the flip angle. *J Magn Reason Imaging*. 2014;40:875-883.

3. Stollberger R, Wach P. Imaging of the active B1 field in vivo. *Magn Reson Med*. 1996;35(2):246-251. doi:10.1002/mrm.1910350217

4. Stupic KF, Ainslie M, Boss MA, et al. A standard system phantom for magnetic resonance imaging. *Magn Reson Med*. 2021;86(3):1194-1211. doi:10.1002/mrm.28779
